# Quality of health service in the local government authorities in Tanzania: a perspective of the healthcare seekers from Dodoma City and Bahi District councils

**DOI:** 10.1186/s12913-023-10381-2

**Published:** 2024-01-16

**Authors:** Richard F. Msacky

**Affiliations:** Department of Business Administration, College of Business Education, P.O Box 2077, Dodoma, Tanzania

**Keywords:** Quality, Healthcare Seekers, Local government authorities, Healthcare Facilities, SERVQUAL Model

## Abstract

**Background:**

Improvement and access to quality healthcare are a global agenda. Sustainable Development Goal (SDG-3) is committed to ensuring good health and well-being of the people by 2030. However, this commitment heavily depends on joint efforts by local authorities and the immediate service providers to communities. This paper is set to inform the status of health service provision in local authorities in Tanzania using the determinants for quality health services in Dodoma City and Bahi District.

**Methods:**

A cross-sectional research design was employed to collect data from 400 households in the Local Government Authorities. The five-service quality (SERVQUAL) dimensions of Parasuraman were adopted to gauge the quality of service in public healthcare facilities. Descriptive statistics were used to compute the frequency and mean of the demographic information and the quality of health services, respectively. A binary logistic regression model was used to establish the influence of the demographic dimensions on the quality of health services.

**Findings:**

The findings revealed that quality health services have not been realised for healthcare seekers. Further, the area of residence, education, and occupation are significantly associated with the perceived quality of health service delivery in the Local Government Authorities.

**Conclusion:**

The healthcare facilities under the LGAs offer services whose quality is below the healthcare seekers’ expectations. The study recommends that the Local Government Authorities in Tanzania strengthen the monitoring and evaluation of health service delivery in public healthcare facilities.

## Introduction

The need for quality service in the health sector has been the focus of global reforms and initiatives to attain Universal Health Coverage [1, 2]. The quality of health service delivery is salient to the satisfaction of the healthcare seekers with the services, utilization of the healthcare facilities, and healthcare performance [3, 4]. Similarly, in Local Government Authorities (LGAs), quality health service delivery creates trust and ownership by the community members of the health service in public healthcare facilities [5]. The public sector’s desire for quality service delivery emerges with the rise of people’s living standards, technology, and awareness that oblige policymakers and decision-makers to shift their focus toward quality services rather than larger quantities of low-quality services [6]. It is equally important to note that the quality of service is a matter of concern in the health sector since negligence in improving healthcare service may cause serious outcomes, including an increase in mortality rate, dissatisfaction with healthcare services, and the community members may reject to utilize the public healthcare services [4, 7]. Thus, countries worldwide, both developing and developed countries, are striving jointly to build health systems to attain quality [2]. The attainment of global development programs, such as SDG-3, which focuses on ensuring good healthcare and well-being of the people by 2030, depends on the quality of services provided by the healthcare facilities [8] under the Local Government Authorities.

To attain quality health service delivery, countries worldwide in the 1980s embarked on reforms that decentralized the service delivery to semi-autonomous institutions such as Local Government Authorities (LGAs) [9]. Similarly, the reforms aimed to provide LGAs and community members more control so they could improve access and quality of service delivery, including healthcare. The reforms also were meant to show that service delivery is planned and provided in preference to the local needs [10–12]. Thus, the reforms resulted in devolution’s decentralization as a strategy for devolving powers, resources, and decision-making to LGAs to improve the quality and governance of services [13, 14].

Like other countries, Tanzania started to implement extensive reform programs as remedial measures for the economic crisis of the late 1970 and 1980 s [13]. The overall objective of the reforms was to ensure quality services within the priority sectors like health and ensure that services conform to the expectations of community members in service provision. The reforms, notably the Health Sector Reforms (HSR), the Local Government Reform Programme (LGRP), the Legal Sector Reform Programme (LSRP), and the Public Financial Management Reform Programme (PFMRP), decentralized service delivery to the LGAs [15].

In Tanzania, the Health Sector Reform and the Local Government Reform Programme of the 1990s greatly concerned quality service delivery in public healthcare facilities. In the governance context, the reforms were implemented in the spirit of decentralization by devolution. The decentralization by devolution involves the transfer of responsibilities and power for services to the local people to promote quality healthcare [14]. In this context, the LGAs (the city or municipality or district, ward, village, or *mtaa*) have clear and legally recognized geographical boundaries over which they exercise authority and within which they can plan and make decisions on service delivery, including health in their area of jurisdiction [16]. Thus, the LGRP and HSR have greatly informed the broad policy of Tanzania Vision 2025, the National Strategy for Growth and Reduction of Poverty (NSGRP), popularly known as MKUKUTA of 2005, and the Tanzania National Health Policy of 2007 [10, 14, 16].

Thus, the LGRP (1998–2008 and 2008–2014) and HSR (1994) resulted in significant organisational, managerial, and financial changes that promoted quality health service delivery in Tanzania from the preference of the healthcare seekers. The changes affected the health services provision and planning at the village, ward, district, region, and national levels. Owing to the LGRP and HSR changes, the health system in Tanzania gave healthcare seekers a more significant say in assessing and monitoring the quality of services through healthcare boards and committees [16]. The outcome of the implementation of the LGRP and HSR was assumed to be the availability of reliable services of high-quality and affordable to the people in healthcare facilities [17]. Similarly, to speed up the LGRP and HSR vision into realisation, Tanzania in 2015 adopted the Health Sector Strategic Plan IV (HSSP-IV) 2015–2020 with a famous slogan for reaching all households with quality healthcare [5]. The HSSP-IV emphasized the availability of drugs, human resources for health, medical equipment, and infrastructure in the dispensaries, health centers, and district hospitals to attain quality service delivery [5, 18]. Both the reforms and strategic plan in Tanzania for the health sector resulted in the empowerment of the district or city council, ward council, village or *mtaa* council in the governance of health service delivery [10, 13, 14, 16, 17].

Studies on healthcare services show that quality healthcare positively impacts satisfaction with service delivery, improved maternal healthcare services, and the utilization of primary healthcare facilities [19, 20]. In the same vein, it is indicated that health service delivery in Dodoma Region of Tanzania is challenged with inadequate human resources, a shortage of essential drugs, and insufficient healthcare facilities for quality service delivery [16, 21, 22]. The challenges in health service delivery tend to influence the choice of healthcare seekers to consume health service delivery based on individual characteristics such as gender, education, marital status, and occupation [23]. A growing body of literature shows that area of residence, gender, age, marital status, and education are essential in judging how consumers perceive the quality of service provided in the facilities [24, 25].

Quality of service can be assessed using the service quality model proposed by Parasuraman, Zeithaml, and Berry. The model uses five dimensions; tangibility, reliability, responsiveness, assurance, and empathy, to explain the experience of quality of service received by the beneficiary (service seeker) in the care facilities [26]. Healthcare seekers refer to the consumer and recipients of health services in a healthcare facility. The consumer and recipient proactively engage with healthcare systems and providers under the LGAs to receive necessary care, whether preventive, diagnostic, curative, or rehabilitative. Service quality assessment from the consumers’ perspective is salient as it helps the providers understand customers’ expectations, perceptions, areas for improvement, and progress made in providing quality services [24, 27].

Similarly, healthcare seekers’ determinants of quality are essential to enable providers to segment healthcare services based on the specific needs of each group in the community. The literature on assessing service quality from consumers’ perspectives is scant in Tanzania; most of the available literature focuses on quality drawing from the experience of health workers [28–31]. Thus, this article partly addressed the gap by explaining the influence of the socio-demographic dimensions on the perceived quality of healthcare service from the experience of the healthcare seekers in the councils of Dodoma City and Bahi District in Tanzania.

## Literature review

The service quality (SERVQUAL) model was developed by Parasuraman, Valerie Zeithaml, and Len Berry to measure the quality of service between 1983 and 1988. The model uses five gaps computed from the discrepancies between expectations and perceptions from five service quality dimensions [3, 26]. The five service quality dimensions are tangibility, reliability, responsiveness, assurance, and empathy [32]. Using SERVQUAL to measure customer expectations and perceptions helps identify opportunities and improve the overall service delivery outcome in the health sector (Butt & Run, 2010). Scholars have widely used the model to make inferences on service delivery quality in the education, business, and marketing sectors [32–35]. Similarly, the model in the health sector is meant to increase the methodological rigor, noting that most studies of health service delivery in the governance context from Tanzania were qualitative [1, 19, 31, 36]. Thus, SERVQUAL dimensions in Table 1 were adopted and modified to determine the quality of service in the healthcare facilities from the perspective of the healthcare seekers in the LGAs of Dodoma City and Bahi District in Tanzania.

**Table 1:** Description of the SERVQUAL Dimensions

	Dimensions	Description
1.	Tangibility	Physical aspects (infrastructure, equipment, personnel)
2.	Reliability	Ability to fulfill what was promised
3.	Responsiveness	Ability to attend and adapt to the needs of the health seeker
4.	Assurance	Competency, courtesy, and credibility to the health seeker
5.	Empathy	Attention and understanding of the health seeker

**Source**: Adopted from Parasuraman, Berry, and Zeithaml, 1994

The use of the SERVQUAL model to assess the quality of health services is significant in that health services in Tanzania were reported to face several challenges [21, 29, 30, 35]. However, the researchers concluded the perception and experience of the health workers [27–30]. The quality assessment for the World Health Organisation emphasized the need for the view of healthcare seekers when assessing the service delivery for sustainability [37]. A study on the quality from the perception of the service seekers is essential as it helps the provider to understand the customer expectations, perceptions, areas for improvement, and progress made in providing quality service [24, 27]. In addition, as the beneficiaries of care, healthcare seekers are critical stakeholders in the health system, and their feedback is necessary to develop responsive, people-centered health delivery systems under the LGAs.

## Research methods and material

### Study design

This study used a cross-sectional research design. A cross-sectional design produces a snapshot of the population under the study at a particular point in time [38]. This design suit studies like this that uses the quantitative approach in the collection and analysis of data to express a relationship in a given situation. Thus, the cross-section design was adopted because the study investigated the existing situation of health services to express the relationship between demographic aspects and the quality of health service delivery. Also, this design was useful in that it could survey 400 heads of the household in time.

### Settings of the study

The study was conducted in the Dodoma City and Bahi District councils, among the LGAs in Dodoma Region, Tanzania. The two councils depict urban and rural LGAs stipulated in the constitution of the United Republic of Tanzania in Article 145, that recognizes establishing the city and district in urban and rural areas to foster service delivery. The LGAs in Dodoma Region were selected because the area is among the regions in Tanzania with critical challenges of service delivery in the healthcare facilities [21, 22, 37]. The challenges include a high maternal mortality rate, a shortage of human resources for health, long waiting times for treatment, and inadequacy of health facilities [21, 22, 39]. Also, Dodoma Region is recently experiencing rapid population growth. This growth has put more pressure on service delivery, including health services. Research shows that population growth significantly impacts service delivery, specifically in healthcare [40]. The National Census of 2022 reveals a tremendous increase in population growth in Dodoma Region at the rate of 3.9% as compared to 2.3 in 2012 [41, 42]. The rapid increase in population growth in the region is associated with the transfer of government offices from Dar-es-Salaam Region to Dodoma Region [22]. Dodoma City and Bahi District councils have many households compared to other councils in the region [41]. Thus, Dodoma City and Bahi District Councils are purposively selected to depict the councils with many households in Dodoma Region to represent urban and rural LGAs in the country. The number of households in the two councils was significant in selecting the unity of inquiry of the study.

Dodoma City has been the capital city of Tanzania since 2016 [22]. It is administratively divided into 41 wards with 170 *mitaa* and 39 villages. The main economic activities of people in Dodoma City are business and farming [21]. The city owns 4 health centers and 27 dispensaries for providing healthcare services to its population. It has the region’s most significant number of households. The Bahi District is among the rural council in the Dodoma Region with the highest number of households. Bahi District Council consists of 22 wards and 59 villages. The main economic activities of the people in Bahi are farming and livestock keeping. The council in its jurisdiction owns 6 health centers and 35 dispensaries [21].

### Description of study participants

The data was collected from the heads of household in Dodoma City and Bahi District Councils. At the household level, the heads of the households were purposively selected as the unit of inquiry. The selection of the heads of the households as the unit of inquiry at the household level was based on the fact that, in most families, the head of the family is in-charge of all the family matters, including seeking health services. The study considered the fact that the head of the household can be a male or female from 18 years of age. The same consideration was made in the previous studies in the context of Tanzania where a person of 18 years was considered an adult person with effective social, political, and economic responsibility of influencing service delivery [10, 13, 14]. Thus, this study considers every healthcare seeker to be 18 years and above as an adult capable of giving information regarding their household and perceived quality. The total population of all the households in the study areas was 92,978 and 49,287 households in Dodoma City and Bahi District, respectively [41].

### Sampling

The study employed a statistical formula by Yamane (1967) to compute the sample size of 400, which was later proportionated to Dodoma City and Bahi District.

$$n=\frac{N}{1+N({e)}^{2}}$$

Where *n* = Sample size, N = Population size, e = The level of precision

$$sample\,size = \frac{{142265}}{{1 + 142265{{(0.05)}^2}}} = 398.9 \approx 400$$

A multi-stage technique was used to select wards, mtaa, or village and household. In the first stage, a list of wards with health centres was obtained from each council. Then, 10 wards with health centres were purposively selected, with 4 from Dodoma City and 6 from Bahi. The second selection involved randomly selecting 2 *mitaa* or 2 villages from each ward with the health centres. Thus, the study, in total, selected 8 *mitaa* and 8 villages. The third stage involved systematic sampling for selecting 400 households using a mtaa or village register obtained from the chairperson of the *mtaa* or village. The head or their representative was interrogated at the household level to provide demographic information and perceived healthcare quality in their village, *mtaa*, wards, or district. The different elements (village, *mtaa*, wards, or district) were selected, given the understanding that the health service delivery in the LGAs in Tanzania is organized into three levels [10]. The lowest level is the dispensary, closely monitored by the village or *mtaa* council. The medium level is the health centre which serves as the referral point for the dispensary and is closely monitored by the ward development committee. The last level is the city or district hospital, at the apex of health service delivery in the LGAs. Thus, the study had to consider all the wards with healthcare facilities assuming that the respondents are likely to have accessed those facilities for healthcare services.

### Data collection and entry

A household survey method in a five-Likert scale questionnaire collected quantitative data from the community members who seek healthcare services in the facilities owned by the Dodoma City and Bahi District Councils. Thus, the community members are referred as health seekers given their proximity to the available dispensaries, health centers and district hospitals. The healthcare seekers were asked about demographic information and perceived healthcare quality using expectations and perceptions of the SERVQUAL dimensions: tangibility, reliability, responsiveness, empathy, and assurance. The challenges of employing expectations and perceptions to assess quality in healthcare is based on the fact that recalling is not always easy and accurate, especially for a community member who visited the healthcare facility in a fragile health situation. To mitigate the challenge of administering questionnaires to ill-community members in the healthcare facility, the study, during data collection, focused on the community members with previous experience of health service delivery under the LGAs in their households. This was decided based on the assumption that all community members were once sick or had accompanied their dear ones to seek healthcare services in the healthcare facilities in their area.

Then, for the purpose of ensuring the validity of the data collection tool especially after translation of the questionnaire into Swahili language and modification of the SERVQUAL model aspects, the tool was reviewed by experts. The experts were the academic staff in the field of public health, business studies, and public administration from the University of Dodoma and the College of Business Education in Tanzania. The experts’ observation helped improve the questionnaire to ensure that it adequately measures the construct of interest. The expert review for ensuring validity was chosen given that it has the potential to identify and correct ambiguities, errors, or biases in research instruments [43].

Thus, the questionnaire captured the respondents’ demographic information, notably their area of residence, gender, age, education, marital status, and occupation. During the actual survey, the study adopted an interviewer-administered questionnaire to generate data from the respondents. The researcher or research assistant read the questionnaire in the Swahili language to the respondents, and the responses were filled in the questionnaire. Then, the Statistical Package for Social Science (SPSS) Version 21 software was employed during data entry and analysis of the quantitative data. The interviewer-administered questionnaire method was proper, given its immediate responses and chances to give clarifications when required. While some of the respondents were from the rural community, the process also helped to do away with the challenges of reading and writing by some respondents.

### Ethics consideration and informed consent

Before the survey, the research assistant was trained in data management and ethics in research.

Debriefing meetings were held each day at the end of the field to identifify difficulties and key emphasize on ethics in research work. The study obtained an approval from Dodoma Region Committee for Medical and Health Ethics. Also, the study obtained oral consent from all the respondents. The oral consent was necessary given that some respondents could not read and write as they did not attend any formal education. Similarly, the respondents were explained that they had the right to withdraw from the study in any moment.

### Data analysis

The study employed a quantitative analysis technique with descriptive and inferential statistics to assess the influence of the socio-demographic dimensions on the quality of service in the health sector. Descriptive statistics were used to compute for frequency and percentages of the healthcare seekers who are consumers and beneficiaries of healthcare services in the LGAs. Similarly, descriptive statistics were used to compute the mean difference scores (P-E) between the perception and expectation dimensions of quality of service. Then, dimension scores with a positive mean difference or zero scores were labeled as high quality of service in healthcare facilities. Likewise, the dimension scores with a negative mean score were regarded as low health service quality in healthcare facilities.

Moreover, for inferential statistics, the Binary Logistic Regression Model (BLRM) was used to predict the effects of healthcare seekers’ determinants on the quality of health services in LGAs. Before the study performed BLRM, a 5 Likert scale points data on the quality of the decentralized health services were transformed into an index scale using the mean score. Later, a dummy variable of quality of service was created using the criteria that the scores greater or equal to the mean score was treated as 0 = High quality and 1 = low quality for scores below the mean. The BLRM was represented using this formula:

$$\log it[\pi (x)] = \log \left( {\frac{{\pi (x)}}{{1 - \pi (x)}}} \right) = {\beta _0} + {\beta _1}{x_1} + \ldots \ldots + {\beta _p}{x_p}$$

Where $\pi (x)$ is the likelihood of the quality health service in the healthcare facilities, *x*_*i*_’*S* are set of independent variables *β*_*i*_’*S* represent the coefficient of a respective independent variable. The findings from the model were presented in a regression in the form of an Unadjusted Odds Ratio (UOR) and Adjusted Odds Ratio (AOR). The term UOR refers to the association of individual predictors on outcome variables without the presence of other variables. The AOR refers to the association of a particular variable, an outcome variable, with the presence of other variables (controlling all other variables). Hence, both unadjusted and adjusted regression analyses were conducted. For the adjusted analysis, it is recommended that all predictors that exhibited a *p*-value of less than 0.2 in the unadjusted regression should be included in the model [44].

The estimated odd ratio was determined by taking the exponent of the regression parameter estimates at a confidence interval (CI) of 95%. The OR shows the increase or decrease in the likelihood of the quality of service delivery in the health facility at a given independent variable level compared to those in the reference category. The reference category was used as a benchmark to which the other group was compared.

## Analysis and findings

### Reliability

Cronbach’s alpha of the five service quality dimensions with expectation and perceptions was computed to determine the internal consistency of the tool. The Cronbach’s alpha scale range from 0 to 1, with a value score above 0.7 representing an acceptable level of internal reliability [38]. As indicated in Table 2, Cronbach’s alpha value above 0.7 indicates a high level of internal consistency for the scale.

**Table 2:** The reliability

Dimension	Expected		Perceived	
	Number of items	Cronbach’s alpha	Number of items	Cronbach’s alpha
Tangibility	3	0.851	3	0.761
Reliability	3	0.829	3	0.860
Responsiveness	4	0.826	4	0.786
Assurance	3	0.817	3	0.869
Empathy	3	0.729	3	0.881

### Demographic characteristics of respondents

The demographic information captured the area of residence, gender, education, marital status, and occupation of the respondents, as presented in Table 3. The data was captured from 400 respondents using a closed-ended questionnaire. Data revealed that 260(65%) and 140(35%) respondents were Dodoma City and Bahi District residents, respectively. The high number of respondents from Dodoma City compared to Bahi resulted from the large numbers of households in Dodoma City that influenced the sampling proportion between the two councils. The large number of households in Dodoma City can be linked to the rapid growth of the population in most cities, which is a common phenomenon in developing countries [42].

**Table 3:** Demographic characteristics of the respondents

Category	Sub-category	Dodoma n(%)	Bahi n(%)	All n(%)
Area of Residence		260(65)	140(35)	400(100)
Gender	Male	151(58.1)	97(69.3)	248(62.0)
	Female	109(41.9)	43(30.7)	152(38.0)
Age	20–35	153(58.8)	57(40.7)	210(52.5)
	36–50	76(29.2)	50(35.7)	126(31.5)
	51 and above	31(11.9)	33(23.6)	64(16.0)
Education	Primary	115(44.2)	99(70.7)	214(53.5)
	Secondary	85(32.7)	26(18.6)	111(27.8)
	College	60(23.1)	15(10.7)	75(18.8)
Marital status	Single	53(20.4)	22(15.7)	75(18.8)
	Married	120(46.2)	83(59.3)	203(50.7)
	Divorce	61(23.5)	23(16.4)	84(21.0)
	Widow	26(10.0)	12(8.6)	38(9.5)
Occupations	Farmer	57(21.9)	75(53.6)	132(33.0)
	Entrepreneur	131(50.4)	49(35.0)	180(45.0)
	Employee	72(27.7)	16(11.4)	88(22.0)

Looking at the gender of the respondents, the findings indicate that the majority of the respondents, 151(58.1%), were males from Dodoma City, and 97(69.3%) were male from Bahi District. The males were the dominant gender in this study, given that the unit of inquiry was the heads of the households. Most African families, Tanzania inclusive, are headed by a man. A woman becomes the head in the absence of the husband in the family. Data from the Tanzania Demographic Health Survey indicates that only one in four households in Tanzania is headed by a woman [45]. Thus, given the household heads distribution based on gender in Tanzania, most families in Tanzania are headed by males.

Likewise, the findings in Table 3 show that 153(58.8%) and 57(40.7%) respondents from Dodoma City and Bahi District were aged between 20 and 35 years respectively. This age size category is an active age group expected to be surrounded by several dependants to support in healthcare, including their children who are still young and elderly parents [14]. Thus, this age group has vast experience visiting healthcare facilities under the LGAs. In the same vein, Table 3 shows that the majority of the respondents 115(44.2%) Dodoma City and 99(70.7%) Bahi District] had a primary level of education as compared to 60(23.1%) and 15(10.7) respondents from Dodoma and Bahi respectively who had college level of education. The findings correspond to the Population and Household Census Report of 2022 that indicated that 83.3% of Tanzanians had primary education compared to the 2.3% who had attained college and university education [42]. The attainment of education acquired by the respondents is expected to influence their understanding and ability to gauge the quality of health services provided in the healthcare facilities under the LGAs.

Also, the findings revealed that 203(50.7%) respondents from both Dodoma City and Bahi District were married. The results are similar to the analysis observed from the census survey report, which established that most household heads, i.e., 50.9%, in Tanzania Mainland, are in a married relationship compared to divorced 2.9% and widow 3.1% [45]. This state of marital status signifies that most respondents are health seekers with responsibilities, including healthcare for their loved ones. Moreover, the findings depict that most of the respondents, 180(45.0%), were entrepreneurs. This is evidence that most healthcare seekers in Dodoma City and Bahi have a reliable income to afford healthcare services in dispensaries, health centres, and district hospitals.

### Quality of health services

The quality of health service was computed from the mean differences between service quality dimensions of perception (P) and expectation (E) (P-E) in Table 4. Five service quality dimensions adopted from SERVQUAL were considered: tangibility, reliability, responsiveness, assurance, and empathy [26]. The study employed the paired t-test to assess the mean difference between the perception and expectation scores of the five service quality dimensions. Interpreting the findings in this section is done based on the set criteria; i.e., the scores dimension with a positive mean difference or zero scores indicates a high quality of health services. Similarly, the dimension score with a negative mean score depicts the low quality of health services. The mean scores for all the SERVQUAL dimensions were negative and significant (Table 4). This negative mean difference score implies that the expectations of healthcare seekers about the quality of healthcare services have not yet been realized. Instead, healthcare seekers experience low-quality health services in healthcare facilities. Thus, the healthcare seekers did not receive optimal care from the available health services under the LGAs.

**Table 4:** The quality of service in the healthcare facilities

S/N	Dimensions	Perceived	Expected	Quality(P-E)	CI(Low-High)	*P*-Value
1	Tangibility	2.37	3.83	-1.46	− (1.33–1.58)	< 0.0001
2	Reliability	2.99	4.03	-1.04	− (1.16-91)	< 0.0001
3	Responsiveness	2.74	4.19	-1.45	− (1.60–1.29)	< 0.0001
4	Assurance	2.92	4.02	-1.10	− (1.22-98)	< 0.0001
5	Empathy	2.86	4.10	-1.24	− (1.36-10)	< 0.0001
**Overall Service Quality Mean**		**2.77**	**4.03**	**-1.26**		

Further analysis was carried out using binary logistic regression in Table 5. As reported in the methodological section, the binary logistic analysis was used to assess the demographic factors associated with the perceived quality of health service delivery in the Dodoma City and Bahi District local councils. The findings show that the perceived quality of health services was significantly associated with the area of residence (*P* = 0.000), education (*P* = 0.008), and occupation (*P* = 0.000). It was observed in Table 5 that the odds of healthcare seekers residing in Bahi had high chances of experiencing low-quality health services compared to those in Dodoma City (AOR 4.019[CI,2.099, 7.695]. Also, the healthcare seekers with secondary (AOR 1.419[CI, 0.728, 2.769] and college (AOR 3.461[CI, 1.385, 8.653] levels of education had high chances of reporting low quality of health services than those with a primary level of education. Similarly, the findings revealed that the odds of experiencing low-quality in health services were high among entrepreneurs (AOR 3.553[CI, 1.823, 6.922] and employees (AOR 1.585[CI, 0.707, 3.556] than those with farming occupations.

**Table 5:** Binary logistic regression results

Variable	Unadjusted (U)		Adjusted (A)	
	OR[95%CI]	*P*-value	OR[95%CI]	*P*-value
**Area of residence**				
Dodoma City	Ref			
Bahi District	6.410[3.574, 11.496]	0.000	4.019[2.099,7.695]	0.000
**Gender**				
Male	Ref			
Female	0.920[0.532, 1.496]	0.764		
**Marital status**				
Single	Ref			
Married	1.219[0.407,3.651]	0.346		
Divorced	0.764[0.299, 1.952]	0.724		
Widow	1.387[0.465, 4.142]	0.574		
**Age**				
20–35	Ref			
36–50	2.348[1.204, 4.579]	0.012	1.018[0.570, 1.818]	0.953
51+	2.348[1.121, 4.915]	0.024	1.130[0.531, 2.406]	0.752
**Education**				
Primary	Ref			
Secondary	0.371[0.151, 0.912]	0.031	1.419[0.728, 2.767]	0.304
College	0.531[0.197, 1.426]	0.210	3.461[1.385, 8.653]	0.008
**Occupation**				
Farmer	Ref			
Entrepreneur	0.355[0.166, 0.762]	0.008	3.553[1.823, 6.922]	0.000
Employee	0.971[0.436, 2.161]	0.942	1.585[0.707, 3.556]	0.263

## Discussions of the findings

This study was set to assess the influence of socio-demographic dimensions on the perceived quality of service delivery in the LGAs of Dodoma City and Bahi. The findings indicated dissatisfaction with the quality of service delivery in the LGAs. Also, the results have revealed that the area of residence, level of education, and occupations were significantly associated with the quality of health service delivery. Taking the area of residence between urban and rural, the study findings revealed that the healthcare seekers in rural councils, including Bahi District, experience more low-quality service delivery. It is noted that rural councils in Tanzania and elsewhere experience more challenges of shortage of medical equipment, drug availability, limited accessibility, shortage of staff houses, shortage of human resources for health, and lack of reliable electricity to support the provision of quality health services [17, 19, 21, 46]. The highlighted challenges to attain quality call for special attention from the central government and Local Government Authorities in Tanzania to develop a strategic plan to speed up rural accessibility, electrification, and housing.

Studies show that in Tanzania, the challenges to shortage of human resources for health are noted in rural councils like Bahi and urban councils of Tanzania [13, 17, 30, 31, 45]. The shortage of human resources for health in Tanzania was 67.9% in 2007 during the launching of the Tanzania Primary Health Service Development Programme (PHSDP) [47]. The PHSDP, among other vital issues, aimed to address the crisis of human resources for health at all levels of health service delivery in the country. The shortage of human resources for health poses a significant challenge in providing quality health services, as accentuated in the Health Sector Strategic Plan IV. Healthcare seekers in rural and urban councils are also constrained by other factors such as distance to the health facilities and economic ability to afford health service delivery, especially out-of-pocket payment for health service seeking [48, 49]. The challenges in the health sector affect the quality of health services provided.

The challenges in the health sector might be linked to the low funds allocated for improving health service quality in the LGAs. For example, in the financial year 2018/2019, the government gave 6.1% of its budget to the health sector. This budget allocation is less than the Abuja Declaration of 2001 agreement, which requires African states to allocate 15% of their national budget to the health sector [5, 50, 51]. Tanzania is a signatory member to this declaration, yet its budget allocation is low to achieve quality health services, as emphasized by Tanzania Health Sector Strategic Plan IV and Tanzania Development Vision 2025, as well as the international commitment, including the SDG. The Health Sector Strategic Plan IV maxim is to provide quality health services to every household in Tanzania [5].

Regarding the level of education, as reported in the findings, the healthcare seekers with secondary and college education were more likely to experience low quality in health services than those with primary education. This finding is congruent with studies conducted in Benin, Ethiopia, and Nigeria [52–54]. The explanation for the results might be attributed to the fact that the more a person gets educated, the more s/he becomes aware of the standards of expected quality in service delivery. It can also be linked to the fact that the more educated healthcare seekers are likely to report low quality of health service because they have been more globally exposed to the local, national, and international service delivery arena. Therefore, such exposure serves as a point of reference from which to judge the quality of services compared to the less educated healthcare seekers.

Similarly, the odds of perceived quality of health as low was higher among healthcare seekers with entrepreneurial and employment occupations than farmers. The explanation for these findings might be attributed to the income obtained from the higher-paying jobs that enable them to access advanced treatment with a shorter waiting time using health insurance. Likewise, those working in the entrepreneurial and employment sectors might have higher health literacy levels, given the nature of their work. The heightened health literacy can lead to more informed decisions and higher expectations regarding quality health services. The findings are supported by the studies conducted in Nigeria and Sweden [54, 55].

The findings presented here should be considered alongside a few noted strengths and limitations. First, the strength of this study lies in the fact that it has the potential to assist health service providers in understanding the healthcare seekers’ expectations, perceptions, areas for improvement, and progress made in providing quality service. Likewise, the study can enable the service providers to segment and plan quality healthcare service delivery based on the significant socio-demographic factors of healthcare seekers, such as area of residence, education, and occupation. Also, this study brings to the attention of stakeholders, namely health service providers, healthcare seekers, policymakers, and government, on the quality of health service in healthcare facilities under LGAs.

Regarding the limitations, this study concentrated on the health services in the two councils of Dodoma Region, and therefore, findings may not reflect the experiences in other councils and regions in Tanzania. Again, the study did not consider the views of service providers, mainly human resources for health, who could have highlighted further issues from the providers’ point of view. Similarly, this study has employed the SERVQUAL model with five dimensions to measure the healthcare seekers’ perceived quality. Using all five dimensions might not provide the sensitivity of each dimension with the demographic characteristics. Despite the limitations, choosing two councils with rural and urban features contributed to bridging the methodological approach and empirical evidence gap.

## Conclusion

Healthcare seekers in the study areas perceive the quality of health services offered in healthcare facilities to be below their expectations. The healthcare facilities under the LGAs offer services whose quality is below the healthcare seekers’ expectations. Further, healthcare seekers in rural councils experience more low-quality service in healthcare than their counterparts in urban councils. Based on the evidence generated in this study, the LGAs should regularly train the health service providers to equip them with more skills and competencies in segmenting health services considering the demographic dimensions. In addition, the study observed that healthcare facilities under the LGAs experience financial and human resource deficits in providing quality services; hence, the community and the government should join hands to curb the deficit. Moreover, the study recommend that future studies can assess the demographic variable with one dimension of SERVQUAL for detecting the sensitive dimension of quality healthcare. Also, the study has mainly employed quantitative analysis techniques to arrive at the conclusion. Using only one method might have overlooked the contextual information that other techniques, such as qualitative, could provide. Thus, future studies can use quantitative and qualitative approaches to understand the phenomena better.

## Data Availability

The dataset is not publicly available; however, upon request the author, it will be made available. The data collection tools are also available on request.
